# Case Report: Importance of MRI Examination in the Diagnosis and Evaluation of COVID-19 mRNA Vaccination Induced Myocarditis: Our Experience and Literature Review

**DOI:** 10.3389/fcvm.2022.844626

**Published:** 2022-04-27

**Authors:** Keita Watanabe, Takashi Ashikaga, Yasuhiro Maejima, Susumu Tao, Mao Terui, Tetsuya Kishigami, Masakazu Kaneko, Ryota Nakajima, Shinichiro Okata, Tetsumin Lee, Tomoki Horie, Masashi Nagase, Giichi Nitta, Ryoichi Miyazaki, Sho Nagamine, Yasutoshi Nagata, Toshihiro Nozato, Masahiko Goya, Tetsuo Sasano

**Affiliations:** ^1^Department of Cardiology, Japanese Red Cross Musashino Hospital, Tokyo, Japan; ^2^Department of Cardiology, Tokyo Medical and Dental University, Tokyo, Japan

**Keywords:** cardiac MRI, COVID-19, mRNA vaccination, myocarditis, case series, review, case report

## Abstract

Acute myocarditis is a rare but serious complication associated with mRNA-based coronavirus disease 2019 (COVID-19) vaccination. In this article, four COVID-19 mRNA vaccination induced myocarditis cases managed at our tertiary Medical Center have been discussed. Three patients had typical myocarditis. One patient suffered from atrioventricular block and heart failure, which required more intensive treatment, but eventually improved. Additionally, a review of cardiac magnetic resonance imaging (MRI) features related to the diagnosis of myocarditis showed that COVID-19 mRNA vaccine-associated myocarditis tend to have more late-gadolinium enhancement (LGE) accumulation in the inferior lateral wall direction. According to a report by the U.S. Centers for Disease Control and Prevention (CDC), the diagnosis of COVID-19 mRNA vaccine-associated myocarditis is based on clinical symptoms, altered myocardial enzymes, cardiac MRI finding, or histopathology. Cardiac MRI is relatively less invasive than myocardial biopsy and plays an important role in the diagnosis of myocarditis. This review may aid in the diagnosis of COVID-19 mRNA vaccine-associated myocarditis.

## Introduction

The Ministry of Health, Labor and Welfare Japan approved a range of coronavirus disease 2019 (COVID-19) vaccines in February 2021, and vaccination has been since then widely promoted by the government through various health education campaigns and initiatives. By the end of November 2021, 79.2% of the Japanese population had received their first dose of the COVID-19 vaccine, and 77.3% had received their second dose (1).

As the younger population started receiving vaccines, adverse events different from those commonly seen in older adults began to occur, including myocarditis. In general, myopericarditis is a very rare adverse event associated with vaccination and has been reported particularly after administration of the smallpox vaccine (2, 3). To the best of our knowledge, in the case of COVID-19, as this is the first time that mRNA vaccines have been used clinically, the current occurrence of post-vaccination myocarditis is of particular concern.

In this study, several cases of myocarditis that were suspected to be associated with mRNA-based COVID-19 vaccination were reviewed, and a literature review has been presented regarding the efficacy and utility of late-gadolinium enhancement (LGE) on cardiac magnetic resonance imaging (cMRI) in the diagnosis of myocarditis.

## Case Presentation

From February to October 2021, four patients with COVID-19 mRNA vaccine-associated myocarditis were admitted to our hospital (Table 1). The diagnosis was based on the definition reported by the U.S. Centers for Disease Control and Prevention (CDC) (4). All patients fulfilled the Lake Louise Criteria (LLC) (5), which is considered as a diagnostic criterion for myocarditis on cMRI.

**Table 1:** Patient demographic and medicals characteristics and associated health outcomes.

	**Case 1**	**Case 2**		**Case 3**	**Case 4**
**Definition**	**Confirmed**	**Confirmed**		**Confirmed**	**Confirmed**
Age, y	19	20		29	48
Sex	Male	Male		Male	Male
Race/ethnicity	Caucasian	Again		Again	Again
**Vaccine type**					
Types of mRNA vaccines	mRNA-1273-Moderna	mRNA-1273-Moderna		mRNA-1273-Moderna	BNT162b2 mRNA-Pfizer-BioNTech
Number of vaccinations	2	2		2	1
History of previous COVID-19 infection	Denied/ negative antigen	Denied/ negative antigen		Denied/ negative PCR	Denied/ negative PCR
**Symptoms**					
Day 1 post-vaccination	Chest discomfort	Fever		Fever	No symptom
Day 2 post-vaccination	Chest pain, pain with breathing, hospital admission	Chest pressure, nausea		Chest pain, hospital admission	Fever, tiredness, diarrhea
Day 3 post-vaccination		Hospital admission			Tiredness
Day 4 post-vaccination					Tiredness
Day 5 post-vaccination					Syncope, tiredness, hospital admission
**Vital signs at presentation**					
Temperature, °C	36.9	39.1		36.2	35.4
Heart rate, bpm	100	106		73	80
Blood pressure, mm Hg	109/58	120/57		117/69	85/57
Respirations, per min	18	20		18	20
Chest x-ray findings	No acute pulmonary disease	No acute pulmonary disease		No acute pulmonary disease	enlarged cardiac shadow
Cardiothoracic ratio (CTR)	48.4%	48.4%		43.4%	54.5%
**ECG findings**					
ST changes	No	ST elevation in V3–6		No	Negative T wave in V4–6
Rhythm	Normal sinus rhythm	Normal sinus rhythm		Normal sinus rhythm	Paroxysmal atrioventricular block
**Echocardiogram**					
Number of days after vaccination	3 days	5 days		3 days	5 days
LV ejection fraction	52	62		58	30
LV end-diastolic internal dimension	48	51		40	47
LV end-systolic internal dimension	35	36		28	36
Intraventricular septal diastolic thickness	9	10		9	14
LV posterior wall thickness	12	10		12	15
E/A	2.09	1.7		1.13	0.63
E/e'	3.68	7.15		4.65	9.02
Regional wall motion abnor- malities	None	None		Non	Diffuse hypokinesis
Diastolic function	Normal	Normal		Normal	
**Cardiac magnetic resonance imaging (cMRI)**					
Number of days between last vaccination and cMRI	5 days	6 days (first time)	45 days (second time)	12 days	12 days
LGE	Sub-epicardial wall of basal-mid infero-lateral LV	Na	mid wall of basal inferior LV	mid wall of anterior, and inferior LV	Mid-wall of basal inferior, Sub-epicardial wall of mid- and infero-septum LV
**Definition**	**Confirmed**	**Confirmed**		**Confirmed**	**Confirmed**
T2WBB high signal	Sub-epicardial wall of basal-mid infero-lateral LV	mid wall of basal inferior LV	Na	mid wall of anterior, and inferior LV	Mid-wall of basal inferior, Sub-epicardial wall of mid- and infero-septum LV
**Laboratory findings**					
**Cardiac troponin I pg/mL**					
Presentation	1,801.7	1,885.6		4,419.6	17,888.7
Peak	5,321.9	5,749		4,419.6	17,888.7
Postdischarge	<10	<10		<10	15.5
CK U/L peak	415	324		154	765
CK-MB U/L peak	30.8	15.3		9	64
WBC	6,500	5,900		6,100	5,000
BNP, pg/mL	9.1	12.1		9	111
CRP, mg/dL	5.63	8.78		1.16	11.32
Coronary angiography findings	ND	MRI negative		CCT negative	CAG no stenosis
**Clinical course**					
Hospitalization duration	5	8		5	11
Treatment(s)	Ibuprofen, ACE-I	Ibuprofen, ACE-I		Ibuprofen, ACE-I	dobutamine, Diuretic, Ibuprofen, ACE-I

*BNP, B-type natriuretic peptide; COVID-19, coronavirus disease 2019; CRP, C-reactive protein; E/A, transmitral Doppler early (E-wave) to late (A-wave) ventricular filling velocities; E/e', E-wave to tissue Doppler early diastolic mitral annular velocity; LGE, late gadolinium enhancement; LV, left ventricular; T2WBB, T2 Weighed black blood; WBC, white blood cell*.

The study was approved by the institutional review board of the Japanese Red Cross Musashino Hospital and was conducted in accordance with the ethical principles of the Declaration of Helsinki as well as with the Japanese Ethical Guidelines for Medical and Health Research Involving Human Subjects. All participants provided their written informed consent for the anonymized publication of these findings and were provided information disclosure documents on our website. The participants were free to opt out of participation at any time without any adverse consequences or the loss of benefits to which they were otherwise entitled.

### Case 1, Case 2, and Case 3

These three cases are relatively similar. Male patients under 30 years of age had developed myocarditis after their second dose of vaccination. They experienced some kind of chest symptoms such as chest pain and chest pressure within a few days after vaccine administration. Electrocardiography changes (ST elevation) were observed only in Case2. Particularly, negative T waves appeared after ST elevation; these negative T waves improved over time. High-sensitivity troponin-I levels were elevated in all cases, and creatine kinase-myocardial band (CK-MB) was also elevated to above the reference level in Case 1 and Case 2. There was no elevation of white blood cell (WBC) and B-type natriuretic peptide (BNP), but C-reactive protein (CRP) levels were elevated in all patients. Cardiac MRI was performed in all patients. LGE was observed in each case with varying localization, and was more common in the sub-epicardial wall. In Cases 1 and Case 3, T2 high signal intensity and LGE were observed simultaneously in the same segment. In Case 2, an examination performed 6 days after vaccination showed only T2 high signal intensity at the sub-epicardial wall of the basal inferior left ventricle. However, an examination performed 47 days later showed LGE in the same area and likewise demonstrated that the T2 high signal intensity had disappeared. Ibuprofen was administered to all patients due to its anti-inflammatory effects. An angiotensin-converting enzyme inhibitor (ACE-I) was administered as well to prevent remodeling.

### Case 4

**Figure 1:**
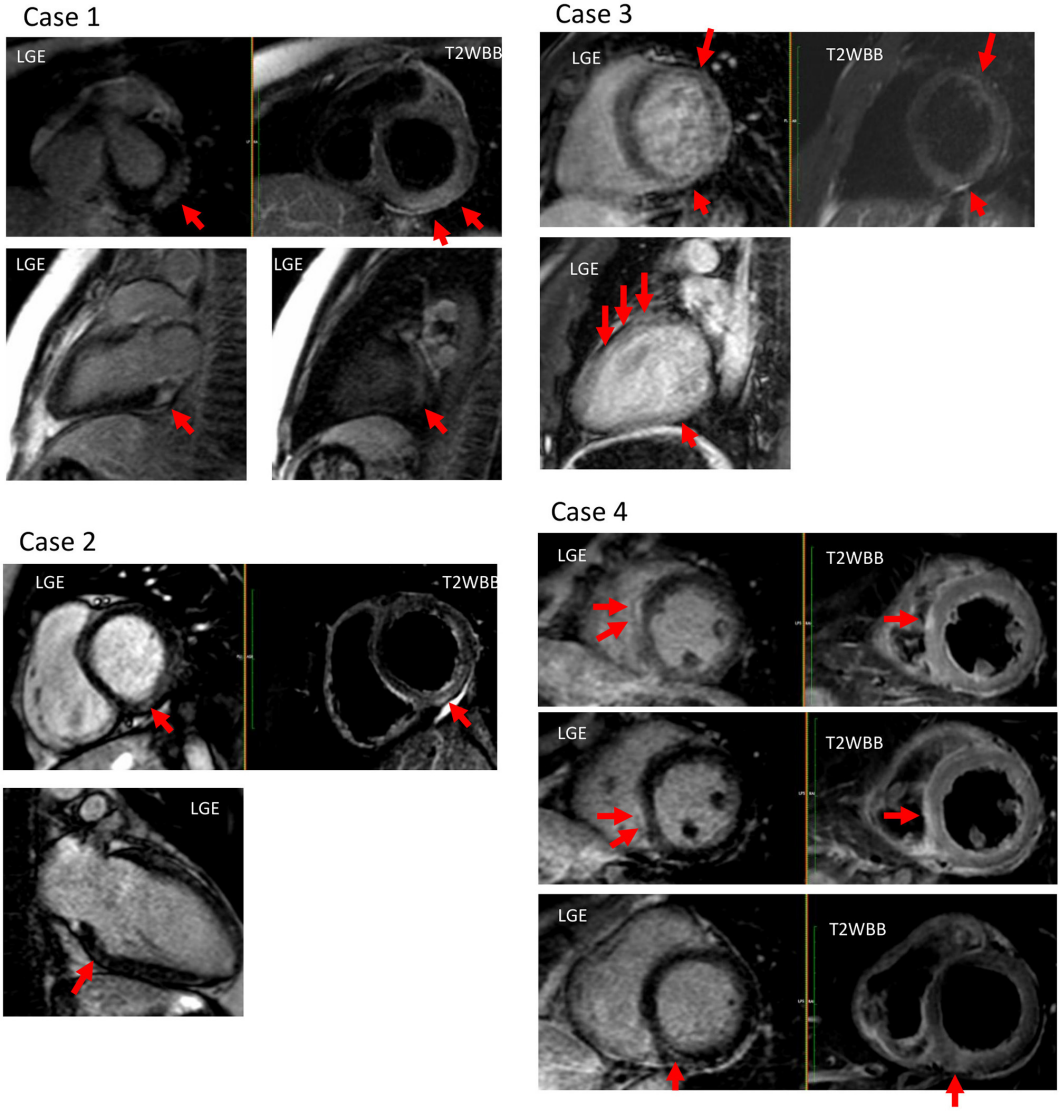
Cardiac magnetic resonance imaging (MRI) of all profiled cases. Case 1: T2 high signal intensity and late-gadolinium enhancement (LGE) were observed at the sub-epicardial wall of the basal-mid inferolateral left ventricular (LV). Case 2: T2 high signal intensity and LGE were observed at the mid wall of the basal inferior LV. Case 3: T2 high signal intensity and LGE were observed at the mid wall of the anterior, and at the inferior LV. Case 4: T2 high signal intensity and LGE were observed at the mid-wall of basal inferior, and at the sub-epicardial wall of the mid- and inferoseptum LV.

Case 4 had a relatively different course compared to the previous three cases. The patient developed fever the day after first dose vaccination, and was referred to the hospital 5 days after vaccination with syncope as the chief complaint. An electrocardiogram revealed paroxysmal atrioventricular block, which was thought to be the cause of syncope. Although a pacemaker lead had to be temporarily inserted for AV block, the paroxysmal AV block resolved 2 days after admission. On echocardiography, marked myocardial hypertrophy and decreased left ventricular contractility were observed. Improvements in hypertrophy and contraction were observed on subsequent echocardiography. In laboratory findings, compared to the previous three cases, BNP levels were elevated, and high-sensitivity troponin-I and CK-MB levels were relatively high. On cardiac MRI, T2 high signal intensity and LGE were observed simultaneously in the mid-wall of basal inferior and sub-epicardial wall of mid-septum and infero-septum left ventricular. Only this patient had an accumulation of LGE and high T2 signal on the left ventricular septum side (Figure 1). Catecholamines (i.e., dobutamine) and diuretics were administered during hospitalization as a treatment for heart failure. Diuretic, ibuprofen, and ACE-I were discontinued following confirmation of negative troponin levels in the outpatient clinic, with no apparent adverse events.

## Discussion

### COVID-19 mRNA Vaccination-Associated Myocarditis

The CDC has recently reported diagnostic criteria for post-vaccination myocarditis (4). The criteria for diagnosis include specific clinical symptoms following vaccination, as well as cMRI findings consistent with myocarditis in the presence of troponin levels above the upper limit of normal and/or histopathologic confirmation of myocarditis. The diagnosis of myocarditis on cMRI is based on the implementation of either the original or the revised LLC (5, 6). We note that cMRI is less invasive than myocardial biopsy and is considered an important diagnostic tool for evaluating vaccine-associated myocarditis.

In this case series, cMRI was performed in all the cases. In Cases 1, 3, and 4, T2 high signals and LGE were observed simultaneously in the same segment, which was considered to fulfill the LLC. In Case 2, T2 high signal intensity was seen on initial examination. Later examination demonstrated the appearance of LGE in the same region. The initial examination showed inflammatory findings, and the LGE observed on the second examination was thought to be the result of fibrosis occurring due to these inflammatory findings. During the observation period, there were cases in which the MRI showed only LGE and no T2 findings, so the LLC could not be fulfilled, and the diagnosis could not be confirmed. More specifically, though these cases had clinical presentations consistent with myocarditis, there are two reasons they were not classified as confirmed cases. First, the quality of MRI was a limiting factor. Namely, the quality of cMRI at our hospital was unacceptable; specifically, the myocardial early gadolinium enhancement ratio could not be evaluated and used parametric mapping techniques with the currently available technology. Thus, findings of sufficient quality might not have been obtained; and may thus, have failed to confirm true myocarditis cases. The second limiting factor is the accuracy of the LLC. Though the LLC present a widely used diagnostic classification system for myocarditis, previous studies have reported that the sensitivity of diagnosing myocarditis when two out of the three main criteria were fulfilled was only ~78% (7). Thus, the accuracy of these criteria alone may not be sufficient to accurately diagnose myocarditis with acceptable sensitivity and specificity. However, studies to date indicate that the modified version of the LLC may provide more accuracy if parametric mapping techniques can be applied (6).

### Cardiac MRI for Myocarditis

Numerous cases of COVID-19 mRNA vaccine-associated myocarditis have been reported till date. cMRI plays an important role in the diagnosis of any form of myocarditis. The American Heart Association (AHA) scientific statement on the management of myocarditis (8) as well as the current European Society of Cardiology (ESC) position statement (9) consider cMRI useful for the evaluation of suspected myocarditis. Japanese guidelines likewise suggest its usefulness (10). More specifically, cMRI provides a non-invasive, biopsy-like approach in order to verify the pathognomonic imaging features associated with and plays a role in the exclusion of myocardial inflammation. The current ESC guidelines on acute and chronic heart failure include a Class I indication for the efficacy of cMRI in the assessment of myocarditis (11). cMRI characteristics of myocardial inflammation may not only aid in the diagnosis of myocarditis but may also provide important and accurate information on prognoses. In acute cases, myocardial edema presenting without LGE on cMRI has been associated with improved recovery and outcomes (12). The relationship between the localization of LGE on cMRI and mortality in myocarditis has been reported as well (13). Thus, cMRI has evolved to become a key evaluation tool in patients with suspected myocardial inflammation.

### Significance of LGE in Myocarditis

LGE is not an essential finding in the original or revised LLC for the diagnosis of myocarditis. However, LGE is the most established technique for detecting myocardial damage (14). The presence of LGE seems to be a good predictor of adverse outcomes in patients with biopsy-proven myocarditis, and has been shown to be superior to other variables in this regard (15). Some recent reports suggest that the location, pattern, extent, and distribution of LGE can stratify the risk for patients with suspected myocarditis. For example, Gräni et al. reported that septal and mid-wall LGE were most strongly associated with major cardiovascular events, including all-cause mortality, worsening heart failure, heart transplantation, and ventricular arrhythmias (16). Aquaro et al. showed that patients with anteroseptal LGE have a worse prognosis than those with LGE at other sites (17). Greulich et al. demonstrated that the presence of mid-wall LGE in the septal segments was associated with a higher long-term mortality rate as compared with the absence of LGE or other LGE patterns in patients with biopsy-proven viral myocarditis (13). One reported mechanism potentially mediating these effects is that the septal LGE might involve the conduction system, thus yielding the substrate for malignant arrhythmias (13). Among the cases presented in this report, only Case 4 revealed LGE in the septal segment. Specifically, Case 4 showed paroxysmal atrioventricular block as a disturbance of the conduction system; this case presentation was relatively severe as compared to the other three cases and thus necessitated intensive treatment. The importance of attaining a greater comprehension of LGE characteristics on cMRI of patients with myocarditis has been emphasized.

### LGE in COVID-19 mRNA Vaccination-Associated Myocarditis

There have been numerous reports of COVID-19 mRNA vaccination-associated myocarditis in recent months; however, adverse reactions are relatively rare and comprehensive reports are limited. Shiyovich et al. reported 15 cases of vaccine-associated myocarditis; and, to the best of our knowledge, it is the largest case series published till date (18). In that report, LGE was observed in 13/15 patients with vaccine-associated myocarditis, and was more common in the inferolateral region.

**Figure 2:**
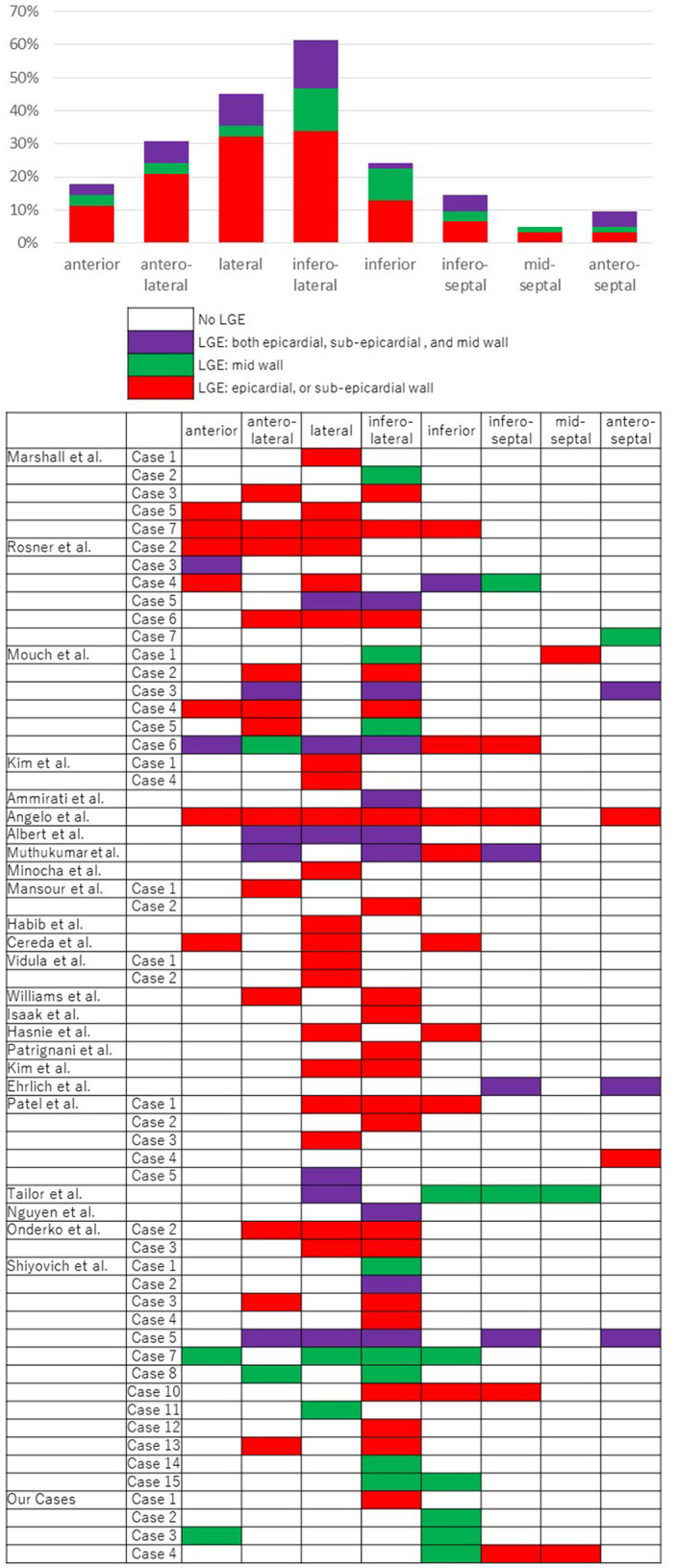
A summary of results regarding late-gadolinium enhancement (LGE) on cardiac magnetic resonance imaging (MRI) for the diagnosis of myocarditis in published cases reports and case series. The localizations were classified as anterior, anterolateral, lateral, inferolateral, inferior, inferoseptal, mid-septal, and anteroseptal. The layers of the myocardium were classified as epicardial or sub-epicardial, mid, and endocardial or sub-endocardial wall.

“PubMed” was mainly accessed and reports were extracted using the keywords “myocarditis,” “mRNA vaccination,” and “COVID-19.” Cases, articles, and review articles without detailed MRI descriptions were excluded. Finally, numerous cases of COVID-19 mRNA vaccination-associated myocarditis presented within 24 publications were reviewed, all of which described the localization of LGE. A total of 62 cases (four cases evaluated by the study authors in the current report, and 58 cases evaluated within previously published case reports and case series) were reviewed in terms of diagnostic imaging, with a focus on LGE findings of cMRI (18–41). The localization was classified as anterior, anterolateral, lateral, inferolateral, inferior, inferoseptal, mid-septal, and anteroseptal. The layers of the myocardium were classified as epicardial or sub-epicardial, mid, and endocardial or sub-endocardial wall. Vertical localization (basal, mid, apical) was not described in many of the cases (i.e., only transverse localization was evaluated). In the present review, the examined cases comprised 61 males and one female with an average age of 29 (±12.4) years. Forty-one patients (66.1%) were under the age of 30 years. Forty-six patients (74.2%) had been vaccinated with the Pfizer mRNA-based vaccine and 16 patients (25.8%) had been vaccinated with Moderna mRNA-based vaccine. Table 2 and Figure 2 summarize the LGE features within cMRI. We found that LGE occurred more frequently on the free wall side (i.e., mainly in the inferolateral region) and occurred relatively less frequently on the septal side. LGE was mostly detected on the epicardia or sub-epicardia; no case with LGE on the left ventricular endocardia was identified.

**Table 2:** Published case reports and case series regarding COVID-19 vaccine-associated myocarditis that describe LGE on cardiac magnetic resonance imaging (MRI).

		**Age**	**Sex**	**Vaccine types**	**Number of vaccinations**	**Time from last vaccination to cardiac MRI**	**LGE: layer**	**LGE: segment**
Marshall et al.	Case 1	16	Male	Pfizer	2nd	NA	Subepicardia	Apical and midchamber lateral wall
	Case 2	19	Male	Pfizer	2nd	NA	Mid wall	Basal inferolateral wall
	Case 3	17	Male	Pfizer	2nd	NA	Subepicardial	Basal anterolateral segment, basal to midventricular inferolateral segments
	Case 5	17	Male	Pfizer	2nd	NA	Epicardia	Anterior and lateral LV
	Case 7	14	Male	Pfizer	2nd	5 days	Subepicardial	Mid and apical left ventricle free wall
Rosner et al.	Case 2	39	Male	Pfizer	2nd	11 days	Subepicardial	Along the anterior and lateral walls
	Case 3	39	Male	Modelna	2nd	5 days	Subepicardial and midmyocardial	Anterior wall
	Case 4	24	Male	Pfizer	1st	7 days	Midmyocardial	Septal and inferior walls
			Male				Subepicardial	Anterior, lateral, and inferior walls
	Case 5	19	Male	Pfizer	2nd	3 days	Multifocal patchy subepicardial and midmyocardial	Lateral and inferolateral walls
	Case 6	20	Male	Pfizer	2nd	6 days	Subepicardial	Lateral, inferolateral, anterolateral walls, apex
	Case 7	23	Male	Pfizer	2nd	3 days	Mid wall	Basal anteroseptal
Mouch et al.	Case 1	24	Male	Pfizer	2nd	NA	Subepicardial	Basal septum
							Mid myocardial	Inferolateral
	Case 2	20	Male	Pfizer	2nd	NA	Subepicardial	Basal and middle anterolateral and inferolateral walls
	Case 3	29	Male	Pfizer	2nd	NA	Diffuse	Basal, inferolateral, anterolateral and anteroseptal walls
	Case 4	45	Male	Pfizer	1st	NA	Subepicardial	Middle anterolateral, inferolateral and apical anterior walls
	Case 5	16	Male	Pfizer	2nd	NA	Midmyocardial	Basal inferolateral
			Male				Subepicardial	Middle anterolateral
	Case 6	17	Male	Pfizer	2nd	NA	Subepicardial	Basal inferolateral, middle inferolateral and infero-septal and apical lateral, anterior and inferior walls
							Mid-myocardial	Middle inferolateral and anterolateral and apical anterior and lateral walls
Kim et al.	Case 1	36	Male	Moderna	2nd	3 days	Epicardial	Apical lateral
	Case 4	24	Male	Pfizer	2nd	3 days	Epicardial, patchy	Lateral
Ammirati et al.		56	Male	Pfizer	2nd	NA	Subepicardial-intramyocardial regions	Basal and apical segments of the infero-lateral wall
Angelo et al.		30	Male	Pfizer	2nd	6 days	Subepicardial	Sparing of the basal and mid-septal segments
Albert et al.		24	Male	Moderna	2nd	5 days	Mid-myocardial and epicardial	Lateral, anterolateral and inferolateral segments
Muthukumar et al.		52	Male	Moderna	2nd	6 days	Midmyocardial and subepicardial	Infero-septal, inferolateral, anterolateral, and apical walls
							Subepicardial	Inferior basal and mesocardial midventricular region
Minocha et al.		17	Male	Pfizer	2nd	NA	Subepicardial	Mid-lateral and apical
Mansour et al.	Case 1	25	Male	Moderna	2nd	6 days	Subepicardial	Anterolateral wall of the mid and apical left ventricle
	Case 2	21	Female	Moderna	2nd	4 days	Subepicardial	Inferolateral wall at the base
Habib et al.		37	Male	Pfizer	2nd	NA	Subepicardial	Basal lateral wall
Cereda et al.		21	Male	Pfizer	2nd	NA	Patchy epicardial	The posterior, anterior, inferior, and lateral walls
Vidula et al.	Case 1	19	Male	Pfizer	2nd	NA	Subepicardial	Basal to mid lateral wall
	Case 2	18	Male	Moderna	2nd	NA	Subepicardial	Mid lateral wall
Williams et al.		34	Male	Moderna	2nd	7 days	Subepicardial	Anterolateral and inferolateral segments
Isaak et al.		15	Male	Pfizer	2nd	NA	Subepicardial	Inferolateral wall
Hasnie et al.		22	Male	Moderna	1st	NA	Subepicardial	Lateral wall and inferior segments at the midventricular and apical LV
Patrignani et al.		56	Male	Pfizer	1st	11 days	Sub-epicardial	Basal and middle segments of the infero-lateral wall
Kim et al.		24	Male	Pfizer	2nd	NA	Sub-epicardial	Basal inferior and inferolateral segment
Ehrlich et al.		40	Male	Pfizer	1st	12 days	Diffuse	Basal and mid anteroseptal and inferoseptal segments as well as in the apical septal segment
Patel et al.	Case 1	22	Male	Pfizer	1st	NA	Subepicardial	Basal inferior, basal inferolateral, and apical lateral
	Case 2	19	Male	Pfizer	2nd	NA	Subepicardial	Basal inferolateral
	Case 3	25	Male	Moderna	2nd	NA	Subepicardial	Lateral
	Case 4	37	Male	Pfizer	2nd	NA	Subepicardial	Basal anteroseptal segment
	Case 5	20	Male	Pfizer	2nd	NA	Subepicardial and mid-myocardial	Basal, mid, and apical lateral segments
Tailor et al.		44	Male	Moderna	2nd	5 days	Mid-myocardial	Mid-septum, infero-septum, and inferior walls at the base to midventricle
							Sub-epicardial and mid-myocardial	Lateral wall at the mid-ventricle and apical lateral wall
Nguyen et al.		20	Male	Moderna	1st	NA	Subepicardial	Mid and basal inferolateral segments
Onderko et al.	Case 2	28	Male	Pfizer	2nd	NA	Epicardium	Apical lateral wall, midanterolateral segments
	Case 3	36	Male	Moderna	2nd	NA	Epicardial	Mid- to distal inferolateral and lateral walls
Shiyovich et al.	Case 1	41	Male	Pfizer	2nd	107 days	Mid-wall	Inferolateral (basal)
	Case 2	24	Male	Pfizer	2nd	103 days	Mid-wall and epicardia	Inferolateral (basal)
	Case 3	17	Male	Pfizer	2nd	7 days	Epicardial	Inferolateral, anterolateral, (basal to apical)
	Case 4	37	Male	Pfizer	1st	48 days	Epicardial	Inferolateral (basal, mid)
	Case 5	39	Male	Pfizer	2nd	8 days	Mid-wall and epicardia	Inferoseptal, anteroseptal (basal), inferolateral, anterolateral (basal), Inferolateral (med), septum, lateral (apical)
	Case 7	19	Male	Pfizer	2nd	43 days	Mid-wall	Inferior (apicalbasal), Inferolateral (mid, basal), anterior (basal, mid), septum, lateral (apical)
	Case 8	28	Male	Pfizer	2nd	139 days	Mid-wall	Inferolateral, anterolateral (basal)
	Case 10	17	Male	Pfizer	2nd	17 days	Epicardial	Inferior, inferolateral (basal), inferior, inferoseptal, inferolateral (mid)
	Case 11	36	Male	Pfizer	1st	63 days	Mid-wall	Lateral (apical)
	Case 12	27	Male	Pfizer	2nd	105 days	Epicardial	Inferolateral (basal)
	Case 13	42	Male	Pfizer	1st	53 days	Epicardial	Inferolateral (apical, basal), anterolateral (basal)
	Case 14	76	Male	Pfizer	2nd	117 days	Mid-wall	Inferolateral (basal)
	Case 15	32	Male	Pfizer	2nd	83 days	Mid-wall	Inferior (basal), inferolateral (basal)
Our Cases	Case 1	19	Male	Moderna	2nd	5 days	Sub-epicardial	Infero-lateral (basal-mid)
	Case 2	20	Male	Moderna	2nd	45 days	Mid-wall	Inferior (basal)
	Case 3	29	Male	Moderna	2nd	12 days	mid-wall	Anterior, inferior wall
	Case 4	48	Male	Pfizer	1st	12 days	Mid-wall	Inferior walls at the base to midventricle
							Subepicardial	Mid-septum, and infero-septum of left ventricular Wall

*LGE, late-gadolinium enhancement; LV, left ventricular*.

Generally, myocarditic infiltrations due to viral infection occur in a peculiar pattern (i.e., predominantly in the lateral free wall, originating from the epicardial quartile of the ventricular wall in myocarditis patients) (42). The patterns of LGE occurring in general viral myocarditis as well as in COVID-19 mRNA vaccination-associated myocarditis appear to be similar.

### Possible Mechanism of COVID-19 mRNA Vaccination-Associated Myocarditis

The mechanisms mediating COVID-19 mRNA vaccination-associated myocarditis have not been elucidated in detail till date. We suspect that the mechanisms mediating COVID-19 mRNA vaccination-associated myocarditis may be similar to those underlying viral myocarditis. Viral myocarditis is mainly due to direct viral damage and the subsequent IL-6-mediated immune response (43).

The mRNA vaccine mainly elicits a local immune response after being injected intramuscularly. However, this immune response is also present systematically, including in the liver, pancreas, and lymph nodes. In experiments among animal models, lipid nanoparticle-modified mRNA influenza vaccines were distributed mainly in the above mentioned organs, but were also detected to a lesser extent in the heart (44). mRNA vaccines do not cause COVID-19, as the mRNA breaks down rapidly in the cell and the vaccine encodes only a portion of the complete virion. Due to the structural design of the mRNA vaccine, it is uncertain whether distribution of vaccine components to the heart could cause direct damage.

The possibility of immunological mechanisms mediating the development of myocarditis following mRNA vaccination needs to be considered. For example, naive T lymphocytes may be primed by autologous proteins released from damaged cardiomyocytes via antigen-presenting cells. In rare cases, it has been reported that this can cause the migration of primed T lymphocytes into cardiovascular tissue, as well as cell-mediated cytotoxicity and lymphocytic myocarditis (45). Pro-inflammatory cytokines are released, increasing T lymphocyte activation and contributing to myocardial damage (46). In various published cases of vaccine-associated myocarditis, myocarditis was found to develop after the second vaccination. We suspect that T lymphocytes primed by severe acute respiratory syndrome coronavirus 2 (SARS-CoV-2) proteins primed by the first vaccination may cause myocarditis. We note that the mRNA-based COVID-19 vaccine is a new vaccine that has not been used previously. More comprehensive elucidation of its pathogenesis is desirable to ensure its safety.

### Report From the Ministry of Health, Labour and Welfare, Japan

In Japan, suspected myocarditis-associated events, including myocarditis and pericarditis, have been reported more frequently among males in the age groups of 10–19 and 20–29 years (3.69 and 9.62 cases per million, respectively, for combined first and second vaccinations for the Pfizer mRNA-based vaccine; and 28.83 and 25.65 cases per million, respectively, for combined first and second vaccinations for the Moderna mRNA- based vaccine) (47). Based on reports of suspected adverse drug reactions in Japan and overseas, the Japanese Ministry of Health, Labour and Welfare had decided to revise the medical package inserts for mRNA vaccines. More specifically, the Ministry considered issuing an alert in light of the high frequency of myocarditis-associated events in young males. The recommendation of the Pfizer-BioNTech mRNA-based vaccine is being considered for males in their teens and twenties, as the frequency of reports of suspected myocarditis-associated adverse events following vaccination with the Moderna mRNA-based vaccine were clearly higher than the frequency of events following vaccination with the Pfizer-BioNTech mRNA-based vaccine. Those who had received the Modena vaccine in the past would be able to choose the Pfizer-BioNTech vaccine later on.

### Clinical Perspective

COVID-19 vaccine-associated cardiomyopathy is frequently reported to develop within 2–3 days following vaccination and presents as chest pain symptomology (44). Elevated myocardial devitalizing enzymes are found in all cases, whereas changes on electrocardiography as well as decreased contraction (left ventricular ejection fraction <50%) on echocardiography have been reported in only 87 and 15% of patients, respectively (44). If chest symptoms are observed following vaccination, it is advisable to consider the possibility of myocarditis and to perform appropriate blood tests and cMRI scans. In the aforementioned CDC report, myocardial biopsy is included as one of the diagnostic criteria for vaccine-associated myocarditis. However, there are few reports regarding relevant pathology findings, likely because the infiltration of inflammatory cells is reduced compared with that in ordinary acute myocarditis; hence, these findings may not lead directly to a definitive diagnosis. There have been many reports on the characteristics of cardiac MRI in evaluating COVID-19 myocarditis, including the current report, and these reports may be more useful and informative in guiding diagnostics and effective clinical decision-making.

In this review, LGE on cardiac MRI was found to be more common on the inferior lateral wall of the left ventricle and relatively less common on the septal side. This finding is similar to existing reports on viral acute myocarditis (14). In viral myocarditis, cases of LGE on the septal side are considered to have a poor prognosis because of its effect on the conduction system of stimulation to the myocardium. In the current review, Case 4, with LGE on the septal side, showed affected atrioventricular conduction and required relatively intensive treatment, and may have the same tendency in COVID-19 vaccine-associated cardiomyopathy. Hence, cardiac MRI may be useful not only for the diagnosis itself, but also with respect to risk stratification. Although most cases occur in young males and the severity of vaccine-associated myocarditis is relatively low in these age groups, cases of cardiac failure have been reported. Thus, based on existing reports, risk stratification should be performed, hospitalization should be considered in some cases, and careful follow-up is always necessary.

## Conclusions

Herein, a report on the detailed features of COVID-19 vaccine-associated myocarditis has been presented. It was found that cMRI is minimally invasive and may aid in the diagnosis of myocarditis. LGE on cMRI tends to occur more frequently on the free wall side and relatively less frequently on the septal side, as in viral myocarditis. These findings can guide future epidemiologic research on this topic of immediate public health importance, directly inform medical guidelines, and help in effective clinical decision-making.

## Data Availability Statement

The raw data supporting the conclusions of this article will be made available by the authors, without undue reservation.

## Ethics Statement

The studies involving human participants were reviewed and approved by Institutional Review Board of the Japanese Red Cross Musashino Hospital. The patients/participants provided their written informed consent to participate in this study.

## Author Contributions

KW, TA, and YM contributed principally to writing the manuscript. ST, MT, TK, MK, RN, SO, TL, TH, MN, GN, RM, SN, YN, TN, MG, and TS revised the manuscript. ST selected the cardiac MRI images and drafted an explanation for the images. All authors contributed to the article and approved the submitted version.

## Conflict of Interest

The authors declare that the research was conducted in the absence of any commercial or financial relationships that could be construed as a potential conflict of interest.

## Publisher's Note

All claims expressed in this article are solely those of the authors and do not necessarily represent those of their affiliated organizations, or those of the publisher, the editors and the reviewers. Any product that may be evaluated in this article, or claim that may be made by its manufacturer, is not guaranteed or endorsed by the publisher.
